# Crohn’s Disease and Ulcerative Colitis: From Pathophysiology to Novel Therapeutic Approaches †

**DOI:** 10.3390/biomedicines12030689

**Published:** 2024-03-19

**Authors:** Dingpei Long

**Affiliations:** 1 State Key Laboratory of Resource Insects, Institute of Sericulture and Systems Biology, Southwest University, Chongqing 400715, China; dplong@swu.edu.cn; 2 Institute for Biomedical Sciences, Center for Inflammation, Immunity & Infection, Digestive Disease Research Group, Georgia State University, Atlanta, GA 30303, USA

## 1. Introduction

Inflammatory bowel disease (IBD) is a non-specific autoimmune condition impacting the gastrointestinal tract, encompassing Crohn’s disease (CD) and ulcerative colitis (UC). The hallmark clinical manifestations of IBD include fatigue, weight loss, malnutrition, the presence of mucopurulent blood in stools and diarrhea, and abdominal pain. This condition follows a chronic course, is challenging to manage, and poses a significant risk of progressing to cancer and eventual mortality. The etiology of IBD is multifaceted, with its pathophysiology remaining elusive. It is well acknowledged that a combination of genetic, environmental, and immunological factors plays a crucial role in the onset of IBD [1].

The objective in managing IBD is to reduce inflammation, achieve and maintain remission, and alleviate the symptoms to improve the patient’s quality of life. Treatment strategies encompass the surgical removal of the affected intestinal sections and pharmacotherapy. Principal pharmacological interventions include aminosalicylates, glucocorticoids, immunosuppressives, and biologics. Despite their effectiveness, these treatments can be expensive and may cause adverse effects, and the disease often relapses. Innovative treatments for IBD are currently being investigated, including stem cell transplantation, fecal microbiota transplantation, and gut microbiota modulation [2,3,4]. Researchers are also exploring targeted therapies for the underlying causes of IBD, such as novel micro- and nanotechnology-based drug delivery platforms [5,6,7].

This Special Issue is dedicated to showcasing the latest research findings on IBD and to offering insights into therapeutic strategies. It compiles five original studies, three review articles, one meta-analysis, and one case report from the fields of biological and medical sciences to discuss the pathogenesis, prevention, diagnosis, and treatment of IBD, providing a comprehensive treatment perspective. The aim of this editorial is not to review each contribution in detail but to invite readers to engage with the articles.

## 2. An Overview of the Published Articles

### 2.1. Criteria for the Assessment and Treatment of IBD

The paramount therapeutic objective for IBD patients is achieving intestinal mucosal healing. Presently, the clinical gold standard for assessing intestinal mucosal healing in IBD patients involves endoscopy and histopathological examination. However, endoscopy, being invasive, time-consuming, and potentially risky, poses acceptance challenges for patients. Consequently, the practicality of utilizing repeated endoscopy for real-time dynamic evaluation of clinical disease activity in IBD patients is limited in clinical settings. Currently, the assessment of disease activity status in IBD patients can be facilitated by biomarkers, including hematological markers such as serum C-reactive protein and fecal markers such as fecal calprotectin (FC) and fecal lactoferrin. These biomarkers are favored by IBD patients due to their non-invasive nature, simplicity, speed, and the objective insight they provide into the clinical disease activity status [8,9,10].

Fecal biomarkers, particularly FC, have emerged as crucial tools for the diagnosis and monitoring of IBD [11]. A systematic review by Bohra et al. [12] (Contribution 1) evaluated the efficacy and reliability of fecal biomarkers in assessing CD by systematically reviewing and meta-analyzing the existing literature to offer clinical practice guidelines. Thirty-three studies that satisfied the inclusion criteria were reviewed, revealing FC’s accuracy in monitoring mucosal healing in CD. Nonetheless, the accuracy of fecal biomarkers was somewhat compromised by the variability in the reference standards and methodologies. Additionally, there was notable heterogeneity in the sensitivity, specificity, and false-positive rates of fecal biomarkers, necessitating further research and validation. In another review, Jucan et al. [13] (Contribution 2) introduced the concept of histological healing (HH) as a therapeutic target in IBD, associated with improved disease prognosis and a decrease in disease-related complications. This study underscores the need for validating and standardizing histological scores to more precisely define micro-activities in clinical practice and trials. It suggests strategies to advance the diagnosis and treatment of IBD by advocating for HH as a therapeutic endpoint, offering a solution to the challenge of persistent inflammation in IBD patients.

### 2.2. Biological Agents and Small Molecule Drug Therapies

The limitations of traditional medications such as glucocorticoids and immunosuppressants in treating IBD, coupled with their side effects from long-term use, have made biologics the leading treatment option as per domestic and international guidelines [14]. Biologics target inflammation by inhibiting various inflammatory mediators or preventing the accumulation of inflammatory cells in the intestines. This category includes agents such as anti-tumor necrosis factor-α, anti-interleukin-12/interleukin-23 (IL-23) antibodies, and anti-integrin antibodies [15,16,17]. These biologics may be administered as monotherapy or in combination with immunomodulators [18]. Nonetheless, challenges such as patient adherence, potential adverse effects, and high costs persist with biologic therapies.

The review by Bretto et al. [19] (Contribution 3) provides an in-depth examination of the latest developments in IBD therapy, covering both novel biologics and small molecule drugs. This includes advancements in more selective Janus kinase inhibitors, anti-IL-23 antibodies, sphingosine-1-phosphate receptor modulators, anti-integrin therapies, and other small molecules under research. Biologics, with their enhanced safety and pharmacokinetic profiles, offer promising mechanisms for modulating cell signaling and leukocyte trafficking. The review also highlights ongoing clinical trials, suggesting that a multi-targeted approach could facilitate personalized IBD treatment strategies in the future, serving as a critical reference for therapeutic decision making in IBD.

Kim et al. [20] (Contribution 4) assessed the efficacy of a combination therapy with methotrexate (MTX) and infliximab (IFX) as an initial treatment for CD in children. The study found no significant differences in clinical, biochemical, endoscopic, and penetrating remission rates between groups after induction therapy and one year of treatment, nor in drug concentrations and anti-drug antibody levels between MTX and IFX. This indicates potential support for combination therapy in pediatric CD, though further large-scale studies are needed for confirmation. Szemes et al. [21] (Contribution 5) investigated the clinical outcomes of using proton pump inhibitors (PPIs) in IBD patients undergoing vedolizumab (VDZ) treatment. The study reported no significant differences in clinical response, endoscopic findings, and clinical remission rates between patients with or without PPIs use during VDZ therapy, highlighting the need for cautious PPIs use in IBD patients. This research provides insights into the combined use of VDZ and PPIs in IBD treatment, offering practical guidance for clinicians.

Opioids are recognized for their role in managing pain and inflammation in conditions such as CD, fibromyalgia, and others [22]. Endogenous opioids increase intestinal permeability and facilitate bacterial translocation, influencing the gut’s microbial balance [23]. Martyniak et al. [24] (Contribution 6) explored the levels of endogenous opioid peptides in CD patients, finding significantly lower concentrations of β-endorphin and proenkephalin compared to the controls across various phases of CD. The study proposes enhancing endogenous opioid peptide secretion as a therapeutic strategy to improve symptoms and quality of life in CD patients, suggesting the potential application of dipeptidyl peptidase 4 inhibitors in CD treatment, thus offering new avenues for clinical management.

### 2.3. Application of Probiotics in the Treatment of IBD

Probiotics, a category of beneficial microorganisms, promote health by colonizing the body and modifying the host’s microbial composition in specific areas. Recent advancements in microbiology have spotlighted the use of probiotics as an innovative approach in treating IBD. Research has shown that probiotics can induce remission in IBD by altering the intestinal microbiota, repairing inflammatory damage to the colonic mucosa, and modulating the microecology of the small intestine [25,26]. Specifically, Lactobacilli prevent pathogenic bacteria from entering and proliferating in the gut by competing for adhesion sites. They also produce short-chain fatty acids and other beneficial substances, thus ameliorating disorders of the intestinal flora. [27]. Najafi et al. [28] (Contribution 7) investigated the impact of various Lactobacillus and Bifidobacterium combinations on the nuclear factor-kappa B (NF-κB) pathway and the secretion of the pro-inflammatory cytokines, interleukin-1 and interleukin-6 (IL-6), in HT-29 cells. Their findings revealed that probiotics significantly attenuated the cellular response to Gram-negative bacteria and decreased the expression of related receptor genes. Moreover, probiotics effectively modulated the NF-κB pathway and diminished the secretion of pro-inflammatory cytokines. This study elucidates the role of probiotics in regulating the Toll-like receptor and NF-κB pathways following stimulation by Gram-negative bacteria, aiming to deepen our understanding of probiotics’ mechanisms in IBD treatment and to establish a foundation for developing novel therapeutic strategies.

### 2.4. Influence of Obesity on IBD

Obesity, recognized as a chronic disease, is witnessing a global surge in prevalence, posing a significant public health challenge. Clinical data and experimental models have indicated the involvement of adipokines in the pathogenesis of autoimmune diseases, with obesity identified as a key environmental factor exacerbating the onset and progression of these conditions [29]. The accumulation of excess macronutrients in adipose tissue triggers the release of inflammatory mediators, such as tumor necrosis factor and IL-6, leading to a pro-inflammatory state and oxidative stress [30]. Kaazan et al. [31] (Contribution 8) reviewed the impact of obesity on IBD, uncovering a correlation between obesity, the incidence and severity of IBD, and an increased risk of surgical complications. The review highlights the intricate relationship between obesity and IBD, marked by chronic inflammation, gut flora dysbiosis, and impaired immune function, which, in turn, influences IBD prognosis. Thus, managing obesity is crucial for the effective treatment and prognosis of IBD patients. Future research should delve deeper into the mechanisms underlying the complex interactions between obesity and IBD.

### 2.5. Other Gastrointestinal-Related Diseases

Rectovaginal fistula (RVF), a passage between the epithelial linings of the rectum and vagina, can be congenital or acquired, presenting as a rare and challenging pelvic floor disorder. In females with CD, CD-related RVF poses significant surgical complexity and a low cure rate, becoming a prominent cause of RVF alongside its growing prevalence [32]. Dimova et al. [33] (Contribution 9) introduced a novel treatment approach for complex RVF, employing a Martius flap combined with micro-fragmented adipose tissue (MFAT) enriched with mesenchymal stem cells. This innovative method has shown success in CD patients, promising significant advancements in regenerative medicine. The study explores MFAT therapy’s effects on the inflammatory response and immune regulation, offering new perspectives on treating anorectal fistula.

Irritable bowel syndrome (IBS), a chronic functional gastrointestinal disorder, is characterized by abdominal pain and altered bowel habits [34]. Tarar et al. [35] (Contribution 10) examined the comorbidity of fibromyalgia syndrome (FM) and chronic fatigue syndrome (CFS) in IBS patients using the US National Inpatient Sample Database. The findings indicate a high comorbidity rate of FM and CFS among IBS patients, with a higher prevalence compared to the general adult population. The study also identified predictors of concurrent FM and CFS in IBS patients, providing valuable insights for clinical care. This research enhances the understanding and management of IBS and contributes to the ongoing investigation into the pathophysiological mechanisms linking IBS, FM, and CFS.

## 3. Conclusions

This Special Issue offers a comprehensive compilation of original research and review articles, spanning a broad spectrum of studies on the prevention, diagnosis, and treatment of IBD conducted by researchers from various disciplines. Our goal is to foster collaboration with scientists globally to explore new therapeutic agents and strategies for IBD and to delve into its pathogenesis. Despite significant advancements, IBD remains incurable at present. The introduction of novel biologics and oral small molecule medications has expanded treatment options for IBD patients. Nevertheless, traditional immunosuppressive drugs are associated with long-term adverse effects, and manipulating the gut microbiota emerges as a promising therapeutic avenue. Looking ahead, there is a pressing need for research into innovative therapeutic drugs and methods to offer more tailored treatment solutions for individuals with IBD.
